# Use of Toothpaste and Toothbrushing Patterns Among Children and Adolescents — United States, 2013–2016

**DOI:** 10.15585/mmwr.mm6804a3

**Published:** 2019-02-01

**Authors:** Gina Thornton-Evans, Michele L. Junger, Mei Lin, Liang Wei, Lorena Espinoza, Eugenio Beltran-Aguilar

**Affiliations:** ^1^Division of Oral Health, National Center for Chronic Disease and Health Promotion, CDC; ^2^DB Consulting Group, Inc., Atlanta, GA.

Fluoride use is one of the main factors responsible for the decline in prevalence and severity of dental caries and cavities (tooth decay) in the United States (*1*). Brushing children’s teeth is recommended when the first tooth erupts, as early as 6 months, and the first dental visit should occur no later than age 1 year (*2*–*4*). However, ingestion of too much fluoride while teeth are developing can result in visibly detectable changes in enamel structure such as discoloration and pitting (dental fluorosis) (*1*). Therefore, CDC recommends that children begin using fluoride toothpaste at age 2 years. Children aged <3 years should use a smear the size of a rice grain, and children aged >3 years should use no more than a pea-sized amount (0.25 g) until age 6 years, by which time the swallowing reflex has developed sufficiently to prevent inadvertent ingestion. Questions on toothbrushing practices and toothpaste use among children and adolescents were included in the questionnaire component of the National Health and Nutrition Examination Survey (NHANES) for the first time beginning in the 2013–2014 cycle. This study estimates patterns of toothbrushing and toothpaste use among children and adolescents by analyzing parents’ or caregivers’ responses to questions about when the child started to brush teeth, age the child started to use toothpaste, frequency of toothbrushing each day, and amount of toothpaste currently used or used at time of survey. Analysis of 2013–2016 data found that >38% of children aged 3–6 years used more toothpaste than that recommended by CDC and other professional organizations. In addition, nearly 80% of children aged 3–15 years started brushing later than recommended. Parents and caregivers can play a role in ensuring that children are brushing often enough and using the recommended amount of toothpaste.

NHANES is a multistage probability sample of the noninstitutionalized U.S. population; data are obtained from assessments made using interview questionnaires and clinical examinations (*5*). This analysis was limited to children and adolescents aged 3–15 years whose parent or caregiver completed the following open-ended questions: “At what age did study participant (SP) start brushing (his/her) teeth?” and “At what age did (SP) start using toothpaste?” The responses were coded into the following four categories: <1 year, 1 year, 2 years, and ≥3 years. Response to the question “How many times (do you/does SP) brush (his/her) teeth in one day?” was recoded into the following three categories: 1 time, 2 times, and 3–6 times. To estimate the amount of toothpaste used, parents were asked, “On average, how much toothpaste (do you/does SP) use when brushing (his/her) teeth?” Responses, based on the amount of toothpaste on the toothbrush, were categorized as smear, pea size, half load, and full load. All analyses were performed using statistical software that accounted for the complex sample design of NHANES. All estimates were obtained using the interview sample weights. Chi-squared tests were used to assess the association between toothbrushing and toothpaste use behaviors and sociodemographic characteristics, and a p-value <0.05 was considered to be statistically significant (*5*).

A total of 5,157 children and adolescents aged 3–15 years were included in this analysis (Table 1). Approximately half (51%) were non-Hispanic white (white), 14.4% were non-Hispanic black (black), and 15.9% were Mexican-American. More than half (52.8%) were from households earning ≥200% of the federal poverty level, and more than two thirds (69.1%) of heads of households had completed more than a high school education. Overall, 20.1%, 38.8%, 26.6%, and 14.5% of children and adolescents were reported to have started brushing their teeth at age <1 year, 1 year, 2 years, and ≥3 years, respectively (Table 2). Approximately 60% of white and black children were reported to have started toothbrushing at age ≤1 year, including 22.9% and 18.6%, respectively, at age <1 year, and 40.8% and 40.0%, respectively, at age 1 year. Among Mexican-American children, nearly half (49.3%) were reported to have started toothbrushing at age ≤1 year, including 15.4% at age <1 year and 33.9% at age 1 year. More than one fifth (22.6%) of Mexican-American children were reported to have initiated toothbrushing at age ≥3 years, compared with 11.4% of white children and 13.9% of black children. Among children living with a head of household with less than a high school education, 44.5% were reported to start tooth-brushing at age ≤1 year compared with 63.2% of those living with a head of household with higher than a high school education. Overall, 60.5% of children aged 3–15 years were reported to brush their teeth twice a day.

**TABLE 1:** Characteristics of a sample of 5,157* children and adolescents aged 3–15 years included in analysis of toothbrushing behaviors — National Health and Nutrition Examination Survey, United States 2013–2016

Characteristic	No.	% (95% CI)
**Age group (yrs)**		
3–6	1,686	29.7 (28.1–31.4)
7–11	2,116	37.7 (36.3–39.2)
12–15	1,355	32.5 (30.7–34.4)
**Sex**		
Male	2,644	51.5 (49.4–53.5)
Female	2,513	48.5 (46.5–50.6)
**Race/Ethnicity**		
White, non-Hispanic	1,333	51.0 (43.2–58.8)
Black, non-Hispanic	1,286	14.4 (10.8–18.8)
Mexican-American	1,119	15.9 (11.8–21.1)
Other	1,419	18.8 (16.0–21.8)
**Poverty status^†^**		
<100% FPL	1,545	23.3 (19.4–27.7)
100%–199% FPL	1,300	23.9 (21.5–26.6)
≥200% FPL	1,882	52.8 (47.1–58.4)
**Head of household education^†^**		
<High school	1,032	15 (12.0–18.7)
High school	939	15.9 (13.5–18.7)
>High school	3,101	69.1 (63.7–73.9)

**Abbreviations:** CI = confidence interval; FPL = federal poverty level.

*Representing an estimated 51,554,933 U.S. children and adolescents aged 3–15 years.

^†^ Excludes 430 children and adolescents with missing values on poverty status and 130 children with missing values for head of household/education level.

**TABLE 2:** Age of initiation of toothbrushing and number of times teeth are brushed per day among children and adolescents aged 3–15 years — National Health and Nutrition Examination Survey, United States 2013–2016

Characteristic	% (SE)				Chi-squared test	% (SE)			Chi-squared test
	Age child initiated toothbrushing					No. of times teeth brushed per day			
	<1 yr	1 yr	2 yrs	≥3 yrs		1 time	2 times	3–6 times	
**Total**	**20.1 (1.1)**	**38.8 (1.2)**	**26.6 (0.8)**	**14.5 (0.9)**	**—**	**34.2 (1.0)**	**60.5 (1.0)**	**5.3 (0.5)**	**—**
**Age group (yrs)**									
3–6	24.7 (1.5)	40.6 (1.3)	25.3 (1.5)	9.4 (1.0)	—*	34.6 (1.7)	59.0 (1.5)	6.4 (0.8)	—*
7–11	19.6 (1.4)	36.9 (1.6)	27.7 (1.6)	15.8 (1.3)		33.7 (1.3)	62.0 (1.3)	4.3 (0.6)	
12–15	16.5 (1.4)	39.3 (2.2)	26.5 (1.8)	17.8 (1.5)		34.3 (2.0)	60.0 (2.0)	5.7 (0.8)	
**Sex**									
Male	19.0 (1.4)	38.9 (1.6)	25.9 (1.1)	16.2 (1.2)	—*	39.1 (1.5)	56.0 (1.4)	4.9 (0.5)	—*
Female	21.2 (1.1)	38.7 (1.4)	27.3 (1.0)	12.8 (0.9)		29.0 (1.2)	65.2 (1.2)	5.8 (0.7)	
**Race/Ethnicity**									
White, non-Hispanic	22.9 (2.0)	40.8 (2.1)	24.9 (1.4)	11.4 (1.3)	—*	38.3 (1.6)	58.5 (1.5)	3.2 (0.5)	—*
Black, non-Hispanic	18.6 (1.8)	40.0 (1.6)	27.4 (1.6)	13.9 (1.6)		34.3 (2.3)	60.1 (2.3)	5.6 (0.7)	
Mexican-American	15.4 (1.4)	33.9 (1.2)	28.1 (1.5)	22.6 (1.7)		26.5 (1.4)	63.8 (1.8)	9.7 (1.2)	
Other	17.5 (1.3)	36.5 (2.1)	29.2 (1.7)	16.7 (1.5)		29.4 (2.0)	63.3 (2.0)	7.3 (0.9)	
**Poverty status**									
<100% FPL	18.0 (1.7)	35.8 (1.6)	27.6 (1.6)	18.5 (1.6)	—*	31.2 (1.5)	60.4 (1.7)	8.4 (1.0)	—*
100%–199% FPL	18.0 (1.7)	39.4 (2.2)	28.8 (1.7)	13.8 (1.7)		34.4 (2.0)	59.9 (2.0)	5.8 (1.0)	
≥200% FPL	23.0 (1.8)	40.1 (1.8)	24.5 (1.5)	12.4 (1.2)		35.9 (1.6)	60.7 (1.6)	3.4 (0.4)	
**Head of household education**									
<High school	9.7 (1.4)	34.8 (2.0)	30.0 (2.1)	25.4 (1.7)	—*	29.0 (2.4)	62.8 (2.5)	8.2 (1.4)	—*
High school	19.3 (1.6)	35.3 (2.6)	28.9 (1.9)	16.5 (1.4)		37.2 (2.8)	54.6 (2.3)	8.1 (1.5)	
>High school	22.6 (1.4)	40.6 (1.6)	25.2 (1.2)	11.6 (1.0)		34.8 (1.3)	61.1 (1.2)	4.1 (0.5)	

**Abbreviations**: FPL = federal poverty level; SE = standard error.

* Statistically significant (p<0.05) associations between toothbrushing patterns and the individual sociodemographic factors.

Initiation of toothpaste use at age <1 year, 1 year, 2 years, and ≥3 years was reported for 9.0%, 35.2%, 32.7%, and 23.1% of children, respectively. Overall, 8.9%, 10.8%, and 7.7% of white, black, and Mexican-American children, respectively, were reported to have started to use toothpaste at age <1 year, whereas 21.4%, 17.3%, and 31.2% of white, black, and Mexican-American children, respectively, were reported to have started at age ≥3 years. Among children living with a head of household with less than a high school education, nearly 6% were reported to have commenced using toothpaste at age <1 year, compared with 10.6% whose head of household had a high school diploma and 9.3% whose head of household had more than high school education (Table 3).

**TABLE 3:** Age child began using toothpaste and amount of toothpaste used while brushing among children and adolescents aged 3–15 years — National Health and Nutrition Examination Survey, United States 2013–2016

Characteristic	% (SE)				Chi-squared test	% (SE)				Chi-squared test
	Age child began using toothpaste					Amount of toothpaste used*				
	<1 year	1 year	2 years	≥3 years		Smear	Pea	Half load	Full load	
**Total**	**9.0 (0.7)**	**35.2 (1.2)**	**32.7 (1.0)**	**23.1 (1.4)**	**—**	**6.5 (0.4)**	**33.4 (1.2)**	**28.7 (0.7)**	**31.4 (1.1)**	**—**
**Age group (yrs)**										
3–6	9.7 (0.9)	39.5 (1.8)	33.9 (1.6)	16.9 (1.5)	—^†^	12.4 (0.8)	49.2 (1.7)	20.6 (1.2)	17.8 (1.3)	—^†^
7–11	9.6 (0.9)	34.4 (1.6)	31.9 (1.3)	24.0 (1.8)		5.1 (0.6)	33.6 (1.6)	32.2 (1.1)	29.1 (1.4)	
12–15	7.7 (1.3)	32.1 (2.1)	32.6 (2.6)	27.6 (1.9)		2.9 (0.8)	18.7 (1.6)	32.0 (1.4)	46.4 (2.0)	
**Sex**										
Male	9.0 (0.9)	33.6 (1.4)	32.3 (1.2)	25.1 (1.8)	—^†^	6.6 (0.6)	33.0 (1.4)	29.1 (1.1)	31.3 (1.4)	NS
Female	9.0 (0.9)	36.9 (1.4)	33.2 (1.3)	20.9 (1.4)		6.4 (0.6)	33.9 (1.5)	28.2 (1.3)	31.5 (1.6)	
**Race/Ethnicity**										
White, non-Hispanic	8.9 (1.3)	36.8 (2.1)	32.9 (1.7)	21.4 (1.9)	—^†^	6.7 (0.7)	37.1 (1.7)	29.0 (1.3)	27.3 (1.4)	—^†^
Black, non-Hispanic	10.8 (1.2)	39.9 (1.7)	32.0 (1.5)	17.3 (1.6)		4.7 (0.5)	24.2 (2.1)	24.7 (1.5)	46.4 (1.8)	
Mexican-American	7.7 (1.0)	29.7 (1.6)	31.5 (2.1)	31.2 (2.7)		7.7 (0.8)	30.0 (1.7)	29.4 (1.5)	32.9 (1.7)	
Other	8.9 (1.1)	31.8 (1.7)	34.0 (1.5)	25.3 (1.7)		6.5 (0.8)	33.5 (1.6)	30.3 (1.5)	29.7 (1.5)	
**Poverty status**										
<100% FPL	10.2 (1.28)	31.7 (1.7)	30.4 (2.0)	27.8 (2.5)	—^†^	7.4 (0.9)	28.0 (1.6)	28.5 (1.8)	36.0 (1.3)	—^†^
100%–199% FPL	8.9 (1.1)	32.6 (1.5)	35.6 (1.7)	22.9 (2.0)		5.7 (0.9)	35.3 (2.2)	25.9 (1.2)	33.2 (2.0)	
≥200% FPL	9.0 (1.2)	38.4 (1.9)	31.9 (1.5)	20.7 (1.5)		6.1 (0.6)	34.9 (1.9)	29.9 (1.3)	29.1 (1.8)	
**Head of household education**										
<High school	5.5 (1.0)	29.4 (1.6)	31.7 (2.6)	33.4 (2.8)	NS	5.7 (0.9)	30.4 (2.3)	29.9 (2.4)	34.0 (1.9)	NS
High school	10.6 (1.4)	31.9 (2.1)	31.6 (1.6)	26.0 (2.2)		6.7 (0.9)	29.9 (2.2)	27.4 (2.3)	36.0 (2.4)	
>High school	9.3 (1.0)	37.2 (1.6)	33.2 (1.3)	20.2 (1.4)		6.5 (0.6)	34.7 (1.4)	28.8 (0.8)	30.0 (1.3)	

**Abbreviations:** FPL = federal poverty level; NS = not significant; SE = standard error.

* Current amount of toothpaste used was based on the amount of toothpaste on the brush reported by parent or caregiver.

^†^ Statistically significant (p<0.05) associations between toothpaste use patterns and the individual sociodemographic factors.

Approximately 60% of children and adolescents aged 3–15 years reported using a half load (28.7%) or full load (31.4%) of toothpaste when brushing. Among children aged 3–6 years, the reported amount of toothpaste varied: 12.4% used a smear, 49.2% used a pea-sized amount, 20.6% used a half load, and 17.8% used a full load (Table 3).

## Discussion

CDC recommends that all persons drink optimally fluoridated water (0.7 mg/L) and if aged ≥2 years, brush their teeth twice daily with a fluoride toothpaste to reduce the risk for dental caries (*1*). CDC also advises parents to consult with their child’s dentist or physician before introducing fluoride toothpaste to children aged <2 years (*6*). The American Academy of Pediatrics (AAP), American Academy of Pediatric Dentistry (AAPD), and American Dental Association (ADA) recommend fluoride toothpaste for all children and limit the amount of toothpaste used by children aged <3 years to a “smear” the size of a grain of rice (*2*–*4*). In this study, >38% of children aged 3–6 years reportedly used a half or full load of toothpaste, exceeding current recommendation for no more than a pea-sized amount (0.25 g) and potentially exceeding recommended daily fluoride ingestion (*1*,*6*). In addition, some children, particularly Mexican-Americans, were reported to have started brushing their teeth and using toothpaste at age ≥3 years, which is later than is recommended. Similarly, some children living in a low-income household or one in which the head of household had less than a high school education were reported to start toothbrushing at age ≥3 years. Recommendations aim to balance the benefits of fluoride exposure for prevention of dental caries with the potential risk for fluorosis when excessive amounts of fluoride toothpaste are swallowed by young children. The findings from this study highlight the importance of recommendations that parents supervise young children during brushing and monitor fluoride ingestion (*7*–*10*).

Recently, CDC and AAP have begun collaborative work to develop messages targeted at pregnant women and new mothers regarding recommended toothbrushing practices. CDC, AAP, AAPD, and ADA recommend that children aged 3–6 years brush their teeth twice daily using a pea-sized amount of fluoride toothpaste. Supervision is emphasized as a critical role for the parent or caregiver as the child first begins using a toothbrush and toothpaste.

The findings in this report are subject to at least three limitations. First, the measures used are based on parents’ self-report, so reporting bias is possible. Second, the question about the amount of toothpaste used focuses on the amount currently used and therefore might overestimate the amount that was used at younger ages. Finally, the type of toothpaste (fluoride versus nonfluoride) was not specified. Use of these self-report questions is part of the CDC Division of Oral Health’s surveillance plan to improve and monitor fluoride exposure. For future surveillance efforts, it would be ideal to know the amount of toothpaste used when the child first started to use toothpaste and to ensure that the parent or caregiver understands the distinction between the amounts of toothpaste recommended for children and adolescents by using visual aids.

The findings suggest that children and adolescents are engaging in appropriate daily preventive dental health practices; however, implementation of recommendations is not optimal. Careful supervision of fluoride intake improves the preventive benefit of fluoride, while reducing the chance that young children might ingest too much fluoride during critical times of enamel formation of the secondary teeth. Health care professionals and their organizations have an opportunity to educate parents and caregivers about recommended toothbrushing practices to ensure that children are getting the maximum preventive effect by using the recommended amount of fluoride toothpaste under parental supervision.

**Summary**

**What is already known about this topic?**

Fluoride prevents dental caries; however, excessive ingestion by young children can discolor and pit the permanent teeth. Toothbrushing should commence when the first tooth erupts, and children aged <3 years and 3–6 years should use a smear the size of a rice grain and a pea-sized amount of toothpaste, respectively.

**What is added by this report?**

In a survey of toothbrushing practices, nearly 80% of children aged 3–15 years began toothbrushing at age ≥1 year, approximately one third brushed once daily, and nearly 40% of children aged 3–6 years used too much toothpaste.

**What are the implications for public health practice?**

Health care professionals can educate parents about using the recommended amount of fluoride toothpaste under parental supervision to realize maximum benefit.
